# Micellization Thermodynamics of Pluronic P123 (EO_20_PO_70_EO_20_) Amphiphilic Block Copolymer in Aqueous Ethylammonium Nitrate (EAN) Solutions

**DOI:** 10.3390/polym10010032

**Published:** 2017-12-28

**Authors:** Zhiqi He, Paschalis Alexandridis

**Affiliations:** Department of Chemical and Biological Engineering, University at Buffalo, The State University of New York (SUNY), Buffalo, NY 14260-4200, USA; zhiqihe@buffalo.edu

**Keywords:** micelle, self-assembly, surfactant, block copolymer, Pluronic, Poloxamer, poly(ethylene oxide), ionic liquid, water, calorimetry

## Abstract

Poly(ethylene oxide)-poly(propylene oxide)-poly(ethylene oxide) (PEO-PPO-PEO) block copolymers (commercially available as Pluronics or Poloxamers) can self-assemble into various nanostructures in water and its mixtures with polar organic solvents. Ethylammonium nitrate (EAN) is a well-known protic ionic liquid that is expected to affect amphiphile self-assembly due to its ionic nature and hydrogen bonding ability. By proper design of isothermal titration calorimetry (ITC) experiments, we determined the enthalpy and other thermodynamic parameters of Pluronic P123 (EO_20_PO_70_EO_20_) micellization in aqueous solution at varied EAN concentration. Addition of EAN promoted micellization in a manner similar to increasing temperature, e.g., the addition of 1.75 M EAN lowered the critical micelle concentration (CMC) to the same extent as a temperature increase from 20 to 24 °C. The presence of EAN disrupts the water solvation around the PEO-PPO-PEO molecules through electrostatic interactions and hydrogen bonding, which dehydrate PEO and promote micellization. At EAN concentrations lower than 1 M, the PEO-PPO-PEO micellization enthalpy and entropy increase with EAN concentration, while both decrease above 1 M EAN. Such a change can be attributed to the formation by EAN of semi-ordered nano-domains with water at higher EAN concentrations. Pyrene fluorescence suggests that the polarity of the mixed solvent decreased linearly with EAN addition, whereas the polarity of the micelle core remained unaltered. This work contributes to assessing intermolecular interactions in ionic liquid + polymer solutions, which are relevant to a number of applications, e.g., drug delivery, membrane separations, polymer electrolytes, biomass processing and nanomaterial synthesis.

## 1. Introduction

Poly(ethylene oxide)-poly(propylene oxide)-poly(ethylene oxide) (PEO-PPO-PEO) block copolymers are commercially available amphiphiles (known as Pluronics or Poloxamers) that have been studied widely and applied in many fields [1,2,3,4,5]. Their ability to self-assemble in solvents [6,7] and interact with particles and surfaces [8] renders PEO-PPO-PEO block copolymers useful amphiphiles for applications ranging from paints and coatings to pharmaceutical formulations [9]. 

The micellization of amphiphilic block copolymers in water has been widely studied [2]. The spontaneous micellization process of Pluronics is entropy-driven and endothermic. Temperature can alter the thermodynamics and structure of Pluronic micellization in water [10]. Following the micellization, water molecules predominantly form hydrogen bonds with the PEO blocks, while the PPO micelle core is mostly dehydrated [11]. At higher temperatures, the hydration of the PEO blocks decreases [12], and the segregation between the PEO and PPO blocks increases [10]. The hydrophobic PPO block plays an important role in the assembly of the core-shell micelle structure [5,13]. The different composition and length of the PEO and PPO blocks of the PEO-PPO-PEO block copolymers lead to varied micelle corona and core sizes and aggregation numbers [14,15]. 

The modification of the solvent properties of water by the addition of electrolytes or polar organic solutes or solvents [16,17,18,19] affects the thermodynamics of PEO-PPO-PEO block copolymer micellization and the micelle structure [20]. Alkali-halide salts act as cosolutes and promote the PEO-PPO-PEO micellization by lowering the critical micelle temperature (CMT) [2]. Some polar organic solvents, such as ethanol and 1-propanol, act as cosolvents that are able to decrease the cohesive forces in the solvent mixture and increase the solubility of the PEO-PPO-PEO block copolymer molecules, resulting in increased critical micelle concentration (CMC). Other solvents, such as glycerol, favor the micellization and decrease CMC due to a contraction (dehydration) of PEO blocks [17], depending on the solvent quality in the mixed solvents [12,16,19,21,22]. 

Ionic liquids (ILs), low-melting salts with combinatorially high chemical diversity, are being considered for applications in various fields [23], such as biomass processing [24,25], pharmaceuticals [26,27], separations and extraction [28,29], catalysis and batteries [23,30] due to their unique properties and intermolecular interactions [23,31]. Some amphiphiles can self-assemble in neat ionic liquids and in ionic liquid aqueous solutions into ordered structures including micelles and lyotropic liquid crystals [31,32,33,34]. The same types of amphiphile self-assembled structures can be formed in select ionic liquids as in aqueous solutions; however, a higher portion of the solvophobic part is frequently required for an amphiphile to form similar structures in ionic liquids due to a greater solubility of hydrocarbons in many ionic liquids [32].

One of the better studied protic ionic liquids, which have available proton(s) on their cations as hydrogen bond donors [32], is ethylammonium nitrate (EAN), which forms an intermolecular hydrogen bonding network similar to water. This provides an opportunity for EAN to replace or be mixed with water in many uses [35]. Homopolymer PEO (molecular weight 38 kDa) can dissolve in EAN with a more contracted conformation (8.1-nm radius of gyration) compared to that in water (9.6-nm radius of gyration), which implies that EAN is a less good solvent for PEO than water [36]. The shell of Pluronic P123 (EO_20_PO_70_EO_20_) spherical micelles shrank by 40% in pure EAN compared to that in water, while the micelle core radii remained similar (1 wt % Pluronic P123 at 25 °C) [37]. The ethyl moiety on the EAN cation leads to the formation of polar and nonpolar segregated domains, which increases the solubility of hydrocarbons and, as a result, can increase the CMC of PEO-PPO-PEO block copolymers [37]. Surface tension measurements [38] of the ionic low-molecular weight surfactant Aerosol-OT (AOT) in EAN indicated a spontaneous, endothermic and entropy-driven micellization process similar to that observed in water [39]. The surface activity of AOT in EAN is weaker than that in water due to the relatively higher dielectric constant and viscosity of EAN [38]. 

Ionic liquids play one or more roles, such as additives and/or solvents, during the self-assembly process in IL-containing water. The role of ionic liquids as additives in decreasing the critical micellization temperature (CMT) of Pluronic F108 (EO_128_PO_54_EO_128_) aqueous solution [40] is mainly affected by the anion and the concentration of the IL [41]. EAN is totally miscible with water and forms hydrogen bonds in the mixture [42]. It was reported that the Pluronic P123 (EO_20_PO_70_EO_20_) lyotropic liquid crystal (LLC) phases formed in EAN and in EAN aqueous solution are similar [43]. 

Whereas there are several studies on PEO-PPO-PEO block copolymer self-assembly in neat EAN [33,34,37,44,45], most have focused on the structure rather than the thermodynamics. Studies on PEO-PPO-PEO self-assembly in aqueous EAN solutions are quite limited and are concerned with concentrated systems that form lyotropic liquid crystals [43]. To our best knowledge, there is no study available on PEO-PPO-PEO block copolymer micellization in aqueous EAN solutions. 

The thermodynamics and structure of PEO-PPO-PEO block copolymer micellization in water are well established [5,13]. The addition of classic salt cosolutes and/or organic cosolvents leads to various effects on Pluronic micellization, depending on the change of solvent quality. While the ionic liquid EAN exhibits interesting and useful properties when mixed with water [46], the lack of research on PEO-PPO-PEO micellization in aqueous EAN solutions leaves a gap in our fundamental knowledge of this self-assembly process. Quantifying the effect of EAN on PEO-PPO-PEO micellization in aqueous solution in terms of thermodynamics and interactions merits further work. 

Isothermal titration calorimetry (ITC) can directly measure enthalpy changes during (physical) mixing or binding or (chemical) reaction; this provides a way to quantify the thermodynamics of PEO-PPO-PEO micellization process in the presence of EAN. ITC has been previously used to study the physicochemical properties and interactions of Pluronic micellization [47,48,49,50]. With relatively large molecular weight and a gradual transition taking place at the CMC [5], the quantification of the thermodynamics during PEO-PPO-PEO block copolymer micellization can be difficult. Moreover, EAN absorbs a large amount of heat when mixing with water [51], which can obstruct the observation of the enthalpy changes caused by PEO-PPO-PEO micellization in such mixtures. To study the IL addition effect on PEO-PPO-PEO micellization in aqueous solution, we designed experiments to exclude the significant enthalpy change resulting from IL dilution by keeping the EAN concentration constant during titration. Moreover, in order to evaluate the interactions between IL, Pluronic and water, we designed a set of ITC titrations to separately determine the IL dilution enthalpy and the enthalpy of IL-Pluronic interactions. Upon subtracting the enthalpy of EAN dilution and of Pluronic interacting with water from the total observed enthalpy during micellization, the enthalpy associated with interactions between EAN and Pluronic has been assessed and is discussed here as a function of Pluronic concentration at varied EAN concentration. In this paper, the thermodynamics (including CMC, free energy, enthalpy and entropy) of Pluronic P123 micellization are determined in EAN aqueous solution with varied concentrations. The effect of EAN is compared to the temperature effect. The interactions within the aqueous EAN Pluronic solution are explored during micellization. This is the first report that quantifies the effect of EAN addition on the change of thermodynamic parameters of PEO-PPO-PEO micellization in aqueous solution. 

## 2. Materials and Methods

### 2.1. Materials

Pluronic P123 poly(ethylene oxide)-poly(propylene oxide)-poly(ethylene oxide) (PEO-PPO-PEO) block copolymer can be represented by the formula EO_20_PO_70_EO_20_ on the basis of its 30 wt % PEO and 5750 g/mol nominal molecular weight. Pluronic P123 was obtained as a gift from BASF Corp. (Vandalia, IL, USA) and was used as received. With a large portion of PPO, Pluronic P123 is relatively hydrophobic. The micellization of Pluronic P123 has a relatively low CMC in water (0.313 mM at 20 °C [13]) and is sensitive to the change of conditions such as temperature [13], which makes this a good system to observe the effects of ionic liquid addition. 

Ethylammonium nitrate (EAN, C_2_H_5_NH_3_NO_3_, melting point = 12 °C) was obtained from Iolitech (Tuscaloosa, Alabama, AL, USA). It was stored in a desiccator and vacuum dried for at least 8 h before use. Milli-Q purified water was used to prepare aqueous solutions. 

Pyrene purchased from Fluka (Buchs, Switzerland) was used to probe the micropolarity of the Pluronic block copolymer-ionic liquid-water systems. Two microliters of 1 mM pyrene in ethanol mixture were added to 3-g solution samples for fluorescence spectroscopy. The resulting pyrene and ethanol concentrations were about 0.7 μM and 6.7 × 10^−4^ vol %.

### 2.2. Sample Preparation

Ionic liquid aqueous solutions: A specified mass of EAN was mixed with a specified volume of water in a volumetric flask (EAN concentration in water ranging from 0 to 3 M), under rolling at room temperature for at least 8 h before use. Each EAN concentration was prepared separately.

Samples for ITC experiments: Pluronic P123 in ionic liquid aqueous solution was prepared by dissolving Pluronic P123 in the ionic liquid aqueous solution prepared as discussed above. These samples were allowed to equilibrate at least 8 h under rolling at room temperature. The samples were measured within two days after preparation.

Samples for fluorescence experiments: 0.1 wt % Pluronic P123 solution was prepared by mixing neat Pluronic P123 with ionic liquid aqueous solution. This stock solution was allowed to equilibrate for around 24 h under rolling at room temperature. Further samples were prepared by diluting this stock solution to achieve Pluronic concentrations in the range 1 × 10^−6^ to 1 × 10^−2^ wt %. The samples were measured within two days following preparation.

### 2.3. Isothermal Titration Calorimetry 

**Figure 1:**
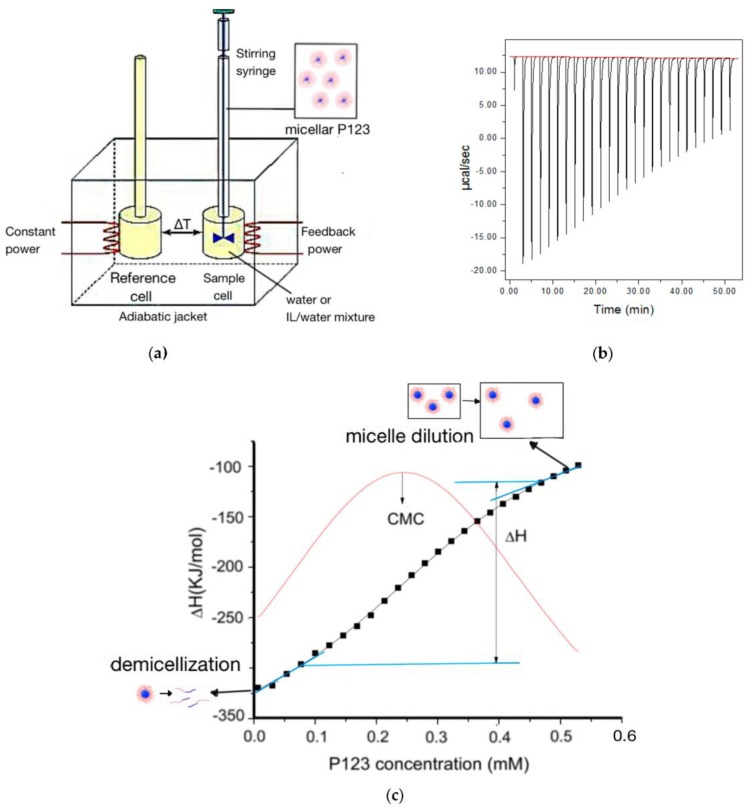
(**a**) Schematic of the titration process for micellization in an isothermal titration calorimeter. Micellar Plurnic P123 inside the syringe is titrated into the sample cell, which contains water or water + ethylammonium nitrate (EAN) solution. ITC records the heat difference between the sample cell and reference cell caused by the dilution and dissociation of injected micelles at constant temperature. (**b**) Raw ITC data of aqueous micellar Pluronic P123 solution titrated into water at 20 °C. (**c**) Enthalpy change during micellar Pluronic P123 solution titrated into water plotted versus Pluronic P123 concentration in the sample cell at 20 °C. The large heat release for the initial injections indicated that all the titrated micelles dissociate into unimers; the relative small heat release over the last injections indicated that the injected micelles only diluted without dissociation. The enthalpy of micellization is determined by the difference between the enthalpy value of extrapolation at the end of the first few injections and the start of the last few injections.

ITC experiments were performed using a Microcal ITC200 calorimeter (Malvern, Worcestershire, UK). (Figure 1) The sample cell (200 μL) was filled with water or water/EAN solution. The syringe (38.4 μL) was loaded with micellar Pluronic P123 in water or in water/EAN solution. Aliquots (1.5 μL per injection) of a micellar P123 solution were injected into the sample cell at 120-s intervals between successive injections. The syringe rotated at a constant speed of 750 rpm. The temperature inside the sample cell was kept constant during the experiment. 

In order to assess the enthalpy changes during the Pluronic micellization process, we successively titrated micellar Pluronic solution into the solvent present in the sample cell. At first, all the Pluronic micelles dissociate into unimers (non-associated block copolymer molecules); but after the Pluronic concentration in the sample cell increased to around CMC, not all titrated micelles dissociate beyond this point. The appropriate experimental parameters (injection volume, interval and titrant concentration) were determined following pre-experiments. We selected the Pluronic P123 titrant concentration at 3.2 mM, so that the P123 concentration in the sample cell changes to span the micellization process as the titration proceeds. We also considered that the heat release/absorption of each titration recorded by ITC should not exceed the maximum measurement capability of the ITC instrument and is not too small compared to the background noise. 

By integrating the enthalpy of each injection and then standardizing by injected amphiphile molar amount, the observed enthalpy change with amphiphile concentration can be obtained. For the first several titrations, the Pluronic P123 concentration in the sample cell is lower than the CMC, and the Pluronic P123 micelles titrated into the sample cell dissociate into unimers. As more injections occur, the Pluronic P123 concentration in the sample cell increases. When the Pluronic P123 concentration in the sample cell reaches the CMC, the magnitude of the observed enthalpy change quickly decreases, indicating that the micellized P123 gradually stops dissociating into unimers. We define the concentration range at which micelles gradually stop dissociating as a “transition region”. For the last few titrations, the Pluronic P123 concentration in the sample cell is well above CMC, the micellar Pluronic P123 no longer dissociates into unimers, and only dilution of the micelles takes place. The micellization enthalpy was calculated as the difference between the enthalpy at the end of the micelle dissociation concentration region and the enthalpy at the start of the micelle dilution region. Extrapolation was conducted by a linear fit of the first/last several points on the enthalpogram. The position where the extrapolation line diverged from the enthalpy curve was designated as the end of the micelle dissociation region/start of the micelle dilution region and was used for the calculation of the micellization enthalpy (as indicated in Figure 1c) [52]. The inflection point of the enthalpogram is assigned as the CMC, which can be calculated by the maximum of the first derivative of the corresponding enthalpy change versus amphiphile concentration [53,54]. In the case of higher temperatures (>22 °C) or higher EAN concentrations (>1 M), the CMC is too small to be quantified by titrating 3.2 mM of Pluronic P123; thus, we used a lower Pluronic P123 concentration (around 1 mM, depending on the experiment) titrant in order to probe a more detailed change at the concentrations around CMC.

### 2.4. Pyrene Fluorescence

A Hitachi 2500 fluorescence spectrophotometer (Tokyo, Japan) was used to record fluorescence emission intensity in the 340 to 460 nm range from pyrene-containing Pluronic P123 solutions at 23 °C. The excitation wavelength was λ = 335 nm. The ratio of the first to the third vibronic peak (*I*_1_/*I*_3_) of the pyrene emission spectrum is a measure of the polarity of the pyrene microenvironment [12]. The micropolarity in EAN aqueous solutions was probed by the *I*_1_/*I*_3_ intensity ratio of the pyrene vibronic band of fluorescence emission spectra. The fluorescence emission of blank (without pyrene addition) EAN and water mixture was measured and used as a control to adjust the baseline. With the Pluronic P123 concentration increase, pyrene partitions into hydrophobic sites (micelle core), which results in a decrease of the *I*_1_/*I*_3_ value due to the lower polarity of a hydrophobic medium compared to that of an aqueous medium. At Pluronic P123 concentrations above the CMC, the value of the *I*_1_/*I*_3_ ratio no longer changes as most pyrene has accumulated in the hydrophobic moieties.

## 3. Results and Discussion

### 3.1. Thermodynamics of Pluronic P123 Micellization in Water

In order to quantify the Pluronic micellization in plain water that serves as a reference system for investigating EAN effects, and to establish the methodology for ITC analysis of Pluronic micellization, micellar Pluronic P123 aqueous solution was successively titrated into water.

**Figure 2:**
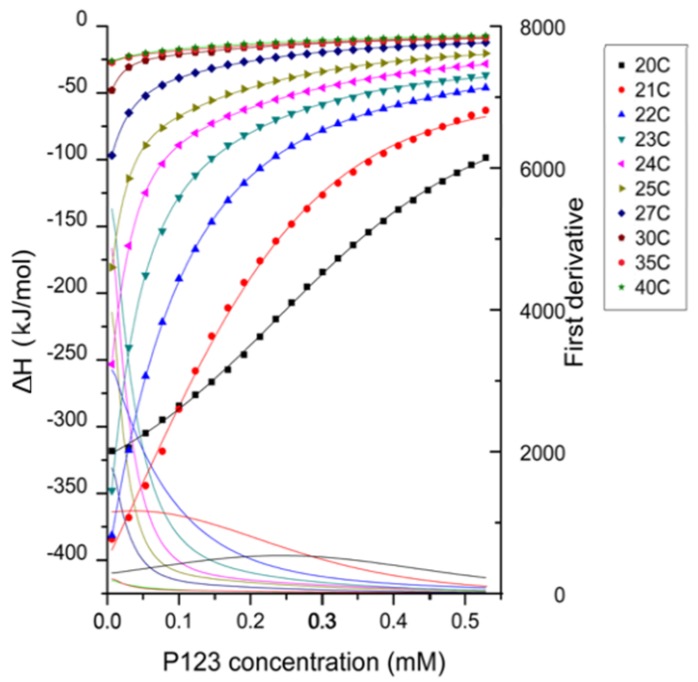
Enthalpy changes generated by titrating 3.2 mM Pluronic P123 in water at varied temperatures (shown in the legend) in the range 20 to 40 °C. The titrant concentration is well above the critical micelle concentration (CMC) (0.313 mM at 20 °C [13]). The enthalpy change for each injection was obtained by ITC and then was standardized by dividing by the amount of the injected Pluronic for each injection. The maximum of the first derivative of the enthalpogram (solid lines) indicates the CMC.

Enthalpy changes recorded during titrations of micellar 3.2 mM Pluronic P123 aqueous solution into water are shown in Figure 2 at various temperatures. The enthalpy change during the whole titration process is negative (exothermic process) at all temperatures considered here. The possible contributions to such exothermic enthalpy profiles include the dilution of micelles and the dissociation of micelles (demicellization) [55].

The enthalpogram has a sigmoidal shape at 20 °C, while it is non-sigmoidal at higher temperatures. This shape transition of the enthalpogram shows that at higher temperatures, the concentration region at which all micelles dissociate into unimers is not observed. As the temperature increased, the CMC and the transition region shifted to lower Pluronic P123 concentration, indicating that higher temperature promoted the micellization process. 

The Gibbs free energy of micellization (Δ*G*_mic_) and the entropy of micellization (Δ*S*_mic_) can be calculated from Δ*H*_mic_ and CMC by the following equations [56]:

Δ*G*_mic_ = *RT*ln(*X*_cmc_)

Δ*G*_mic_ = Δ*H*_mic_ − *T*Δ*S*_mic_

where *X*_cmc_ is the amphiphile concentration (mole fraction) at the CMC.

**Figure 3:**
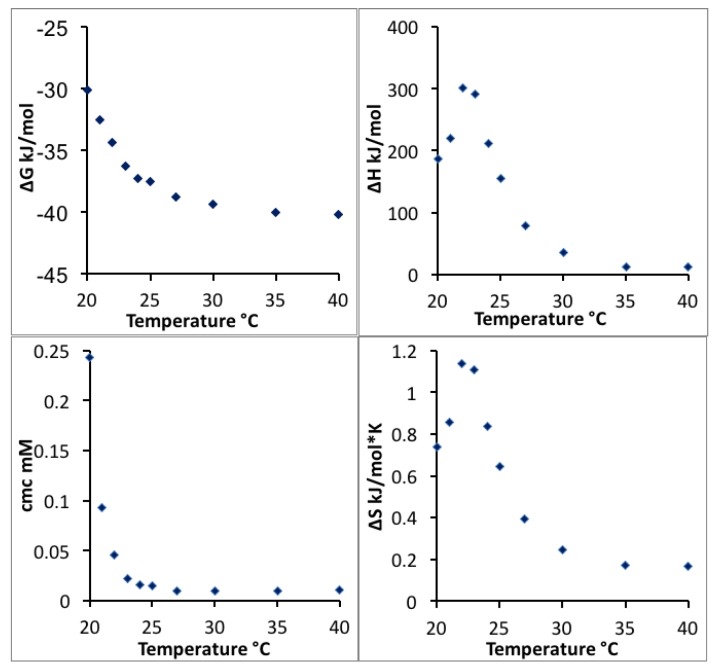
Micellization free energy, enthalpy, CMC and entropy of Pluronic P123 in water plotted as a function of temperature.

The CMC, enthalpy, Gibbs free energy and entropy of micellization extracted and calculated from the enthalpograms in Figure 2 are plotted as a function of temperature in Figure 3. The more negative free energy of micellization values at higher temperatures reflect that Pluronic P123 molecules are thermodynamically easier to assemble into micelles at higher temperature, consistent with previous findings [13]. The positive value of micellization enthalpy Δ*H*_mic_ over the entire temperature range considered here indicates that Pluronic P123 micellization in water is endothermic. The positive Δ*S*_mic_ observed at all the temperatures considered here contributes to the negative free energy and drives the micellization. At lower temperatures (20 to 22 °C), the entropy increase plays an important role to drive Pluronic P123 micellization; while at higher temperatures (>22 °C), the decrease of Δ*H*_mic_ primarily contributes to the decrease of Δ*G*_mic_ and further lowers the CMC. It is noted that at temperatures above 35 °C, the enthalpy of micellization decreased to a very small value according to ITC measurements. These enthalpy values may be caused by the micellization region shifting to very low Pluronic concentrations at this temperature, and the extrapolation method (described in Section 2) is not precise due to the steep change of enthalpy during the initial few injections. The change of micellization enthalpy with temperature is in agreement with studies reporting an increase of micellization enthalpy at lower temperature and a decrease of micellization enthalpy at higher temperature [57]; however, the lowest micellization enthalpy reported in aqueous solution was around 60 kJ/mol at a CMT of 60 °C [58]. The decrease of micellization free energy with temperature increase can be attributed to the temperature-dependent difference in the solvation of PEO and PPO blocks in aqueous solutions. With increasing temperature, PPO blocks experience a large degree of dehydration [59]. The ITC technique provides quantitative information on the change of enthalpy and entropy as the temperature increases. The micellization enthalpy is often assumed to be independent of temperature [13]. However, this assumption is not proper for the temperature-sensitive PEO-PPO-PEO block copolymers.

**Figure 4:**
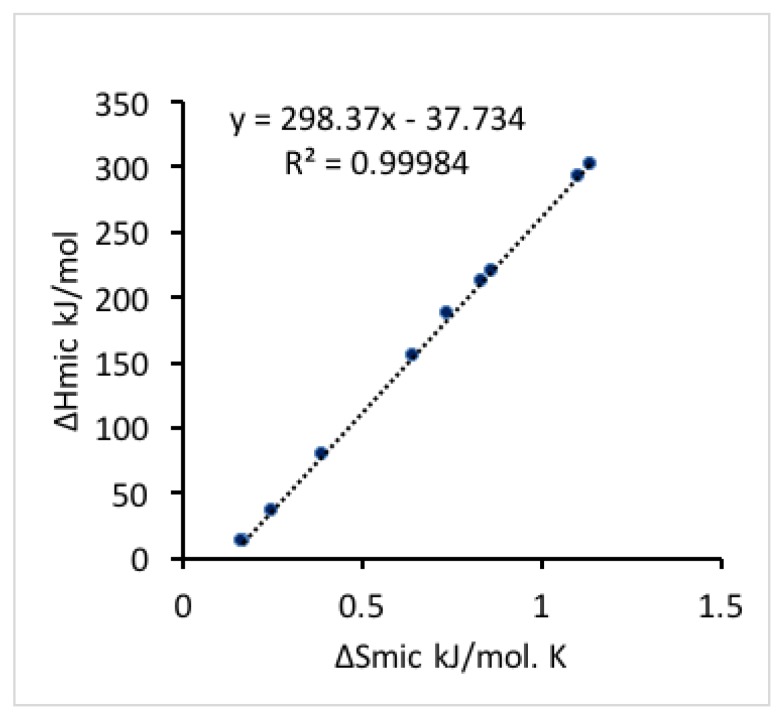
Enthalpy-entropy compensation of Pluronic P123 micellization process. The slope is defined as the compensation temperature (298.37 K) characterizing solvent-solute interaction.

As the temperature increases, the micellization entropy and enthalpy change in the same trend, i.e., they both either increase or decrease together. A linear relationship between the micellization enthalpy and entropy at varied temperature (shown in Figure 4) indicates that changes in Δ*H*_mic_ are compensated by changes in Δ*S*_mic_ for the micellization process [52], which keeps the free energy negative and results in a spontaneous process. The slope of the linear regression of micellization enthalpy versus entropy provides the compensation temperature, which characterizes solvent-solute interactions [60]. The compensation temperature determined here is 298.4 K, which is close to the compensation temperature reported for other Pluronics block copolymers in aqueous solutions characterized by ITC and surface tension (298.1 K for Pluronic 127 (EO_100_PO_65_EO_100_) [52], and 294.9 K for Pluronics F88 (EO_104_PO_39_EO_104_) and F68 (EO_79_PO_28_EO_79_) [57]).

### 3.2. EAN Effects on Pluronic P123 Micellization in Aqueous Solutions

**Figure 5:**
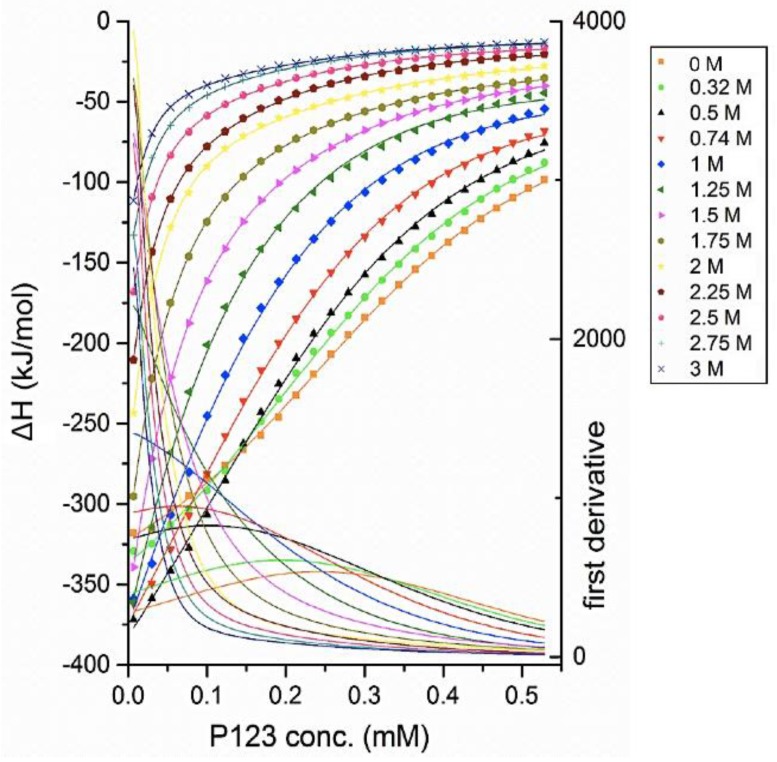
Enthalpy changes of titrating micellar 3.2 mM Pluronic P123 in EAN aqueous solution into aqueous solution with the same EAN content at 20 °C. The enthalpy change for each injection was obtained by ITC and then was standardized by dividing by the amount of the injected Pluronic for each injection. The maximum of the first derivative of the enthalpogram (solid lines) indicates the CMC. The EAN concentration at each titration is shown in the legend.

Having characterized PEO-PPO-PEO micellization in aqueous solution with ITC, we assess here the effect on Pluronic micellization of EAN addition to water. The enthalpy changes of titrating micellar Pluronic P123 in (EAN + water) into (EAN + water) solution were measured by ITC. (Figure 5) Significant heat is absorbed upon mixing EAN and water [51], which indicates an energetically unfavorable (endothermic) mixing resulting from the reduction of strong electrostatic attractions and hydrogen bonds between the EAN ions in the presence of water. Because the large heat absorption during EAN dilution cannot be ignored, it would hinder the assessment of the “real” effect of the EAN presence on Pluronic P123 micellization in aqueous solution. 

To study the EAN effect on Pluronic P123 micellization, 3.2 mM Pluronic P123 in EAN aqueous solution was successively titrated into EAN aqueous solution (having the same EAN content as in the titrant). By keeping the EAN concentration the same in the titrant as in the sample cell, the significant heat change caused by possible EAN dilution during the titration can be excluded, and the results thus obtained reflect the actual enthalpy change of Pluronic P123 micellization in EAN + water mixture. Various EAN concentrations were used in order to assess the effect of EAN concentration on PEO-PPO-PEO micellization. 

The observed enthalpy change is negative during all the titration process with EAN present at varying concentrations, suggesting that the EAN presence does not alter the exothermic aspect of Pluronic P123 demicellization in aqueous solutions. With higher EAN concentration, the CMC significantly shifted to a lower P123 concentration. For example, upon 1 M EAN addition to water, the CMC is 0.06 mM, much lower than the 0.24 mM CMC in plain water. From the enthalpograms of Figure 5, the CMC and micellization enthalpy were extracted through the inflection point and the extrapolation respectively (discussed in Section 2). The Gibbs free energy and entropy of micellization were then calculated based on the value of CMC and micellization enthalpy. Δ*H*_mic_ of Pluronic P123 are measured over the EAN concentration range 0 to 3 M; however, other parameters related to CMC can only be assessed at EAN concentrations up to 2 M. At higher EAN concentrations, the CMC is too low to be measured with this experiment, as the micelles no longer completely dissociate into unimers even at the very first injection. The inability to obtain the enthalpy of micelle dissociation makes it difficult to accurately determine the micellization enthalpy at EAN >2 M. By extrapolating the enthalpy change of the first few injections to infinite dilution (zero Pluronic concentration), an estimation of the micelle dissociation enthalpy was obtained and then used to calculate the micellization enthalpy. With an EAN concentration higher than 3 M, the heat release from each injection is too small to be accurately determined, but the trend from the experiments reported here indicates that more concentrated EAN leads to lower micellization enthalpy.

**Figure 6:**
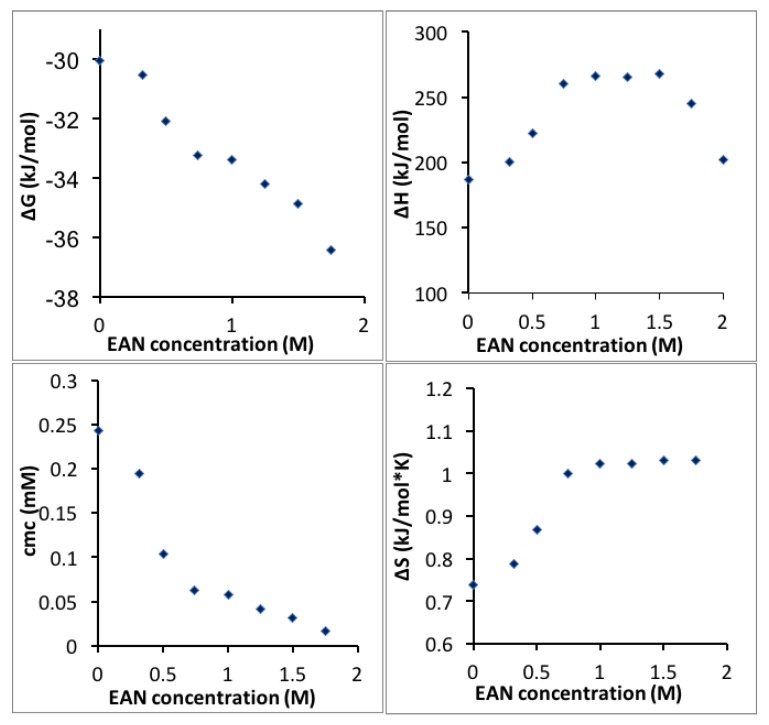
Δ*G*_mic_, Δ*H*_mic_, Δ*S*_mic_ and CMC of Pluronic P123 micellization in EAN aqueous solution plotted as a function of EAN concentration.

The CMC, Gibbs free energy, enthalpy and entropy of Pluronic P123 micellization in EAN aqueous solution are plotted as a function of EAN concentration in Figure 6. The Gibbs free energy of Pluronic P123 micellization decreased monotonically with increasing EAN concentration in water, suggesting that EAN promotes Pluronic P123 micellization in aqueous solution, which is similar to the effect of increasing temperature. For example, the addition to water of 1.75 M EAN lowers the CMC to the same extent as a temperature increase from 20 to 24 °C. The entropy of micellization increased with EAN addition. This can be rationalized in terms of the water molecules arranged in the proximity of the Pluronic hydrophobic chains being released into the bulk solution and becoming more disorganized during micellization. However, when the EAN concentration is higher than around 1 M, the micellization entropy no longer increases significantly with EAN concentration. The properties of the EAN and water mixture depend on the ratio of EAN and water. At dilute EAN aqueous solution (<0.02 mole fraction, 1 M, 10.6 wt % EAN), water molecules solvate around the ions of EAN, which behaves like a typical 1:1 electrolyte in water [61]. However, at higher EAN concentrations, distinct semi-ordered nano-domains of EAN and water have been reported by molecular dynamics simulation even though EAN and water are completely miscible in the macro-scale, which is different from traditional ion-water interactions [62]. Concentrated EAN aqueous solutions exhibit different properties, e.g., lower conductivity, from dilute EAN aqueous solutions [63]. As the EAN concentration in water increases, instead of EAN being released into the bulk during micellization, EAN forms ion clusters, which hinder the entropy increase [64]. With higher EAN concentration in water, the enthalpy of micellization decreases further to contribute to a lower free energy and facilitate micellization.

**Figure 7:**
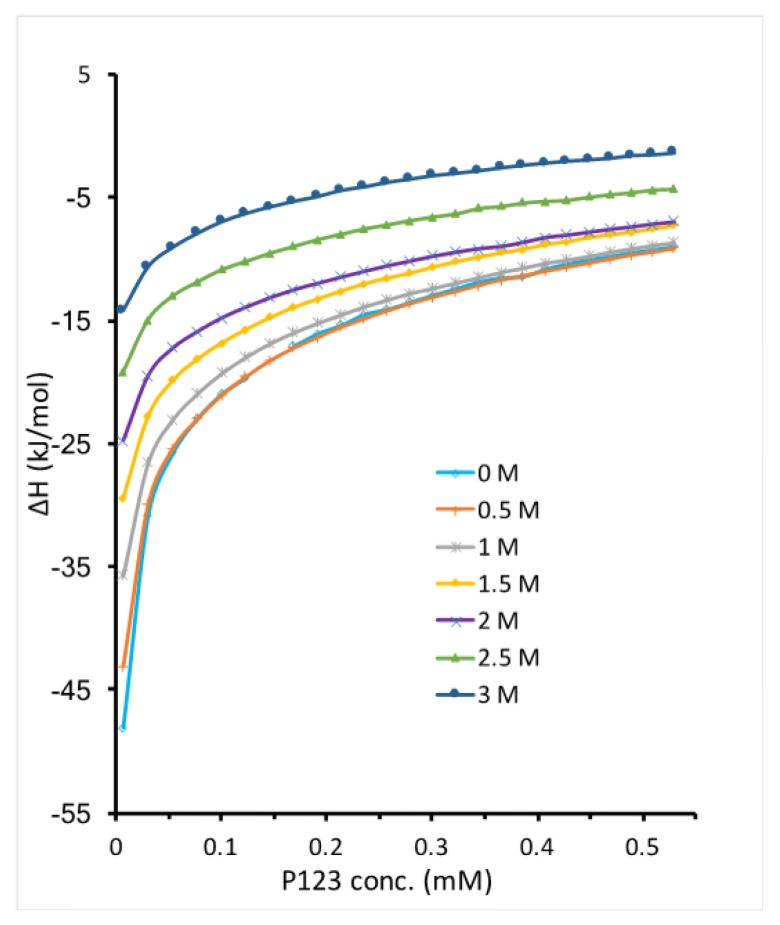
The enthalpy change of titrating 3.2 mM Pluronic P123 in EAN aqueous solution into EAN aqueous solution with the same content at 30 °C. The EAN concentration in each experiment is shown in the legend.

Both temperature increase and EAN addition promote Pluronic P123 micellization by disrupting the water solvation around PEO-PPO-PEO molecules. While the interactions between EAN and water can be affected by temperature, it is interesting to see how EAN addition affects Pluronic micellization at a higher temperature. We titrated micellar Pluronic P123 in EAN and water mixture into the same EAN and water mixture at 30 °C. The enthalpy change during titration shows that the addition of EAN at 30 °C further lowers the micellization enthalpy compared to the cases of either only increasing temperature or adding EAN to water (Figure 7). At the same concentration of EAN, the micelle formation region shifted to a lower Pluronic P123 concentration at 30 °C. For example, the CMC of Pluronic P123 with 0.5 M EAN addition is 0.12 mM at 20 °C, which shifted to a concentration much lower than 0.1 mM at 30 °C. This is in agreement with the literature suggesting that the hydrogen bonds between IL and hydrophilic block weakened as temperature increased and lead to enhancement of solvatophobic interactions and consequently decrease CMC [53]. The enthalpy of Pluronic P123 micellization in EAN aqueous solution can be determined by the extrapolation method described in Section 2. However, the very small micellization enthalpy (less than 30 kJ/mol) determined in this way may not be accurate due to the inability to determine the enthalpy of micelle dissociation as discussed in Section 3.1.

**Figure 8:**
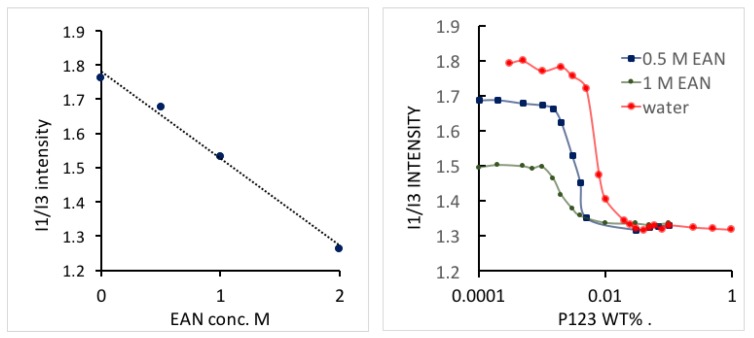
(**Left**) Pyrene fluorescence emission *I*_1_/*I*_3_ intensity ratio of EAN aqueous solutions; (**right**) pyrene fluorescence emission intensity *I*_1_/*I*_3_ ratio plotted against Pluronic P123 block copolymer concentration in aqueous solutions having different concentration of EAN.

The polarity change in amphiphile solutions can reflect the solvent conditions as they affect the process of micelle formation. In order to measure the polarity of aqueous EAN Pluronic solution during micellization [12], pyrene fluorescence spectroscopy experiments were conducted for various concentrations of Pluronic P123 in EAN aqueous solutions. The higher the *I*_1_/*I*_3_ intensity ratio, the stronger the polarity of the medium is. We noticed that the *I*_1_/*I*_3_ intensity ratio of the solvent decreased linearly as with the EAN concentration, due to the less polar nature of EAN compared to water. The *I*_1_/*I*_3_ intensity ratio rapidly decreased with the Pluronic P123 concentration increase, caused by pyrene preferentially locating in the hydrophobic micelle core (Figure 8). The micropolarity in the micelle core did not change with EAN addition, suggesting that EAN does not partition in the PEO-PPO-PEO micelle core. CMC was determined by the breaking point of *I*_1_/*I*_3_ ratio from dramatically decreasing to reaching a plateau at higher Pluronic P123 concentration. CMC values obtained from pyrene fluorescence are consistent with the CMC values obtained from the analysis of ITC presented in Figure 6. To our best knowledge, there is no report available on pyrene fluorescence in EAN aqueous solutions. 

### 3.3. Interactions between PEO-PPO-PEO Block Copolymer and EAN during Micellization

We evaluated in Section 3.2 the change of thermodynamic parameters of PEO-PPO-PEO block copolymer micellization in aqueous solution with EAN addition. We found that the micellization entropy and enthalpy increase at lower EAN concentrations and then decrease at higher EAN concentrations, which indicated that the interactions within this system may change as the EAN concentration increases. This three-component system comprises water, Pluronic and EAN; within this system each two components can interact and compete with the other component. It is reported that EAN behaves differently in water at varied concentrations [63]; the intermolecular interactions between EAN and PEO-PPO-PEO molecules were assessed during Pluronic micellization in pure EAN [37]. However, the interactions operating between EAN and Pluronic in aqueous solutions during micellization are still not established. 

**Figure 9:**
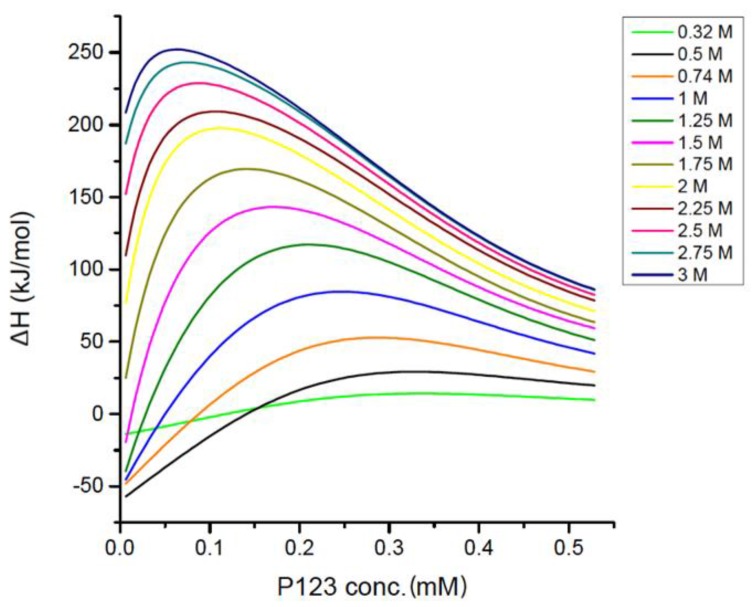
Enthalpy of Pluronic P123 and EAN interaction during micellization plotted as a function of Pluronic concentration. The interaction enthalpy is obtained by subtracting the EAN dilution enthalpy and the enthalpy of Pluronic in water from the observed enthalpy during titrating micellar Pluronic in water into EAN aqueous solution. The titrations were conducted at varied EAN concentrations as indicated in the legend.

To assess the intermolecular interactions between PEO-PPO-PEO and EAN, we designed titration experiments as follows. First, we successively titrated aliquots (1.5 μL) 3.2 mM micellar Pluronic P123 in water into EAN aqueous solution (first titration), for which the observed enthalpy includes the enthalpy of EAN dilution in water and that of Pluronic interaction with EAN and water. Then, we kept all the other conditions the same and titrated the same amount (1.5 μL per injection) of water into EAN aqueous solution (second titration) in order to capture the enthalpy caused by EAN dilution. By subtracting the enthalpy of EAN dilution (second titration) from the total observed enthalpy from the first titration, the enthalpy caused by IL dilution in water can be excluded. By comparing the adjusted micellization enthalpy with that from titrating micellar Pluronic P123 into water (obtained in Section 3.1), we have a way to assess the enthalpy of EAN and Pluronic interactions [65,66] (Figure 9). Other researchers have used a similar method to probe intermolecular interactions between two components within a three- or more component system [67], for example, the effect of surfactant addition on Pluronic micellization [68]. 

In this analysis, we assume that the total observed enthalpy change (Δ*H*_obs_) of titrating (Pluronic + water) into (water + EAN) equals the summation of the enthalpy of mixing between each of two components:

Δ*H*_obs_ = Δ*Η* [EAN-water] + Δ*Η* [EAN-P123] + Δ*Η* [water-P123]

However, during the actual PEO-PPO-PEO micellization in EAN aqueous solution, the three components interact simultaneously. The neglect of three-component interactions in the above-mentioned assumption might raise issues for accurate calculation of the Pluronic-EAN interaction enthalpy. Still, the trend of interactions between EAN and PEO-PPO-PEO during micellization at varied EAN concentration can be established in this way. 

Below the CMC, EAN interacts with PEO-PPO-PEO unimers in the sample cell, and the interaction enthalpy increases with Pluronic P123 concentration. Above the CMC, EAN interacts with both unimers and micelles, the enthalpy of EAN-Pluronic interactions increases to a lesser extent and is further weakened by the dilution of EAN during titration. The maximum of the interaction enthalpy shifts to lower P123 concentration with increased EAN presence, which is the same trend as that of CMC. In this titration method, the EAN concentration in the sample cell is not constant during the titration; instead, EAN was diluted as more P123 aqueous solution was titrated into the sample cell. At the end of the titration, EAN was significantly diluted, which explains the decrease of the interaction enthalpy at the last several injections. 

Different types of interactions in colloidal systems are not all additive. In colloidal systems, when we discuss interactions at distances smaller than tens of nanometers, the sizes of the colloidal particles are comparable to such small distances, and the discreteness of the material matters [69]. However, we can still obtain valuable insights of the interactions affecting the micellization process from the above discussed enthalpy change during P123 micellization. The exothermic process of Pluronic P123 micelle dilution and micelle dissociation in aqueous solution suggests a formation of water-Pluronic hydrogen bonds during the solvation of the Pluronic micelles and unimers. The presence of EAN affects Pluronic P123 micellization through electrostatic interactions and hydrogen bonding with water and with PEO-PPO-PEO molecules. It is known that the enthalpy of EAN mixing with water is endothermic [63], which indicates bond-breaking of the cation-anion interaction and of the water molecule network during the formation of water-EAN complexes. At lower EAN concentrations below CMC, the negative enthalpy of EAN-Pluronic interaction indicates the formation of hydrogen bonds between EAN and Pluronic unimers. It was reported that EAN is able to form a network of hydrogen bonds with PEO, which promotes segregation between PPO and PEO blocks [70]. The positive enthalpy (endothermic) of EAN-Pluronic P123 interactions during micelle formation (at higher Pluronic concentrations) indicates an enthalpy-unfavorable process, which may be due to a break of EAN-Pluronic hydrogen bonds and release of solvent into the bulk solution. The measured positive enthalpy of EAN-Pluronic interactions at higher EAN concentrations suggests that the addition of EAN disrupted the Pluronic-water interaction, resulting in a decrease of the fraction of hydrated methyl groups and consequently lowering the CMC. The dehydration effect of EAN on Pluronic aqueous solution is similar to a classic salt co-solute [66]. As the EAN concentration increases, the enthalpy of Pluronic-EAN interactions increases significantly (up to around 250 kJ/mol), which can explain the increase of the total observed enthalpy (from −375 to −110 kJ/mol for the first injection, shown in Figure 5) upon EAN addition.

## 4. Conclusions

The micellization thermodynamics of a representative Pluronic PEO-PPO-PEO amphiphilic block copolymer are evaluated here in the presence of the common protic ionic liquid ethylammonium nitrate (EAN). Isothermal titration calorimetry (ITC) experiments have been designed to obtain the thermodynamic parameters (including enthalpy, free energy and entropy) of Pluronic P123 (EO_20_PO_70_EO_20_) micellization in EAN aqueous solution, from which the interactions between each component in this system can be assessed. The thermodynamic parameters of Pluronic P123 micellization in water at varied temperature are also examined as a reference system.

The presence of EAN promotes PEO-PPO-PEO block copolymer micellization in aqueous solution (with CMC for Pluronic P123 decreasing from 0.24 to 0.016 mM upon addition to water of 1.75 M EAN at 20 °C) through an increase in the entropy and/or decrease in enthalpy of the system, resulting in lower Gibbs free energy. The addition of EAN has a similar effect on micellization with increasing temperature. It is noted that at EAN concentrations lower than 1 M, the enthalpy and entropy of Pluronic P123 micellization increased with EAN concentration, whereas the enthalpy and entropy both decreased at EAN concentrations higher than 1 M, which can be attributed to EAN forming semi-ordered nano-domains in water. 

The change of interactions between EAN and PEO-PPO-PEO during micellization at varying EAN concentrations was accessed through subtracting the EAN dilution enthalpy from the observed micellization enthalpy. We consider EAN as an additive and as a co-solvent, as it interacts with water and PEO-PPO-PEO molecules at the same time, which reflects the unique properties of ionic liquids during the micellization process. The observed enthalpy of Pluronic-EAN interactions suggests bond formation and breaking between EAN, Pluronic and water. EAN can also be viewed as a cosolvent that performs similar to glycerol in worsening the solvent condition and promoting micellization when added to water. 

Our findings on the effects of EAN on PEO-PPO-PEO micellization in aqueous solution share similarities with the findings on the effect of salt addition. It would be interesting to explore further the similarities and differences between ionic liquids and classic salts on Pluronic micellization. 

In this study, ionic liquid effects on amphiphilic block copolymer micellization thermodynamics in aqueous solution have been assessed through directly-measured enthalpy changes during this micellization process. The enthalpy of PEO-PPO-PEO–EAN interactions is reported for the first time. This improved understanding benefits applications concerning self-assembled or colloidal systems. The intermolecular interactions within self-assembly can guide the design of materials with improved properties. The tuning of micellization thermodynamics can benefit drug delivery with the selection of appropriate additives and solvents in use for nano-carrier assembly and disassembly. This work contributes to assessing the intermolecular interactions in ionic liquid-polymer solutions, which can be applied in biomass processing, polymer electrolytes [71] and membrane separations [72].
